# The influence of weight and gender on intestinal bacterial community of wild largemouth bronze gudgeon (*Coreius guichenoti*, 1874)

**DOI:** 10.1186/s12866-016-0809-1

**Published:** 2016-08-22

**Authors:** Xuemei Li, Qingyun Yan, Einar Ringø, Xingbing Wu, Yongfeng He, Deguo Yang

**Affiliations:** 1 Key Laboratory of Freshwater Biodiversity Conservation, Ministry of Agriculture of China Yangtze River Fisheries Research Institute, Chinese Academy of Fishery Sciences, No.8, 1st Wudayuan Road, Donghu Hi-Tech Development Zone, Wuhan, Hubei 430223 China; 2 Key Laboratory of Aquatic Biodiversity and Conservation of Chinese Academy of Sciences, Institute of Hydrobiology, Chinese Academy of Sciences, Wuhan, China; 3 Norwegian College of Fishery Science, Faculty of Biosciences, Fisheries and Economics, UiT The Arctic University of Norway, N-9037 Breivika, Norway

**Keywords:** *Coreius guichenoti*, Intestinal microbiota, Fish gender, Yangtze River

## Abstract

**Background:**

Largemouth bronze gudgeon (*Coreius guichenoti*) is of economic importance in China, distributed in upstream regions of the Yangtze River in China. But it has recently dramatically declined and is close to elimination. However, there is little knowing about the character of its intestinal microbiota. This study was conducted to elucidate the intestinal microbiota of wild largemouth bronze gudgeon with different body weight and gender.

**Results:**

Thirty wild largemouth bronze gudgeon were measured for body length and body weight, and identified for male and female according to gonadal development, and thereafter the intestinal microbiota’s were assessed by MiSeq sequencing of 16S rRNA genes. The results revealed that phyla Proteobacteria and Tenericutes were dominant in wild largemouth bronze gudgeon intestine independent of the body weight. Shannon’s and Inverse Simpson’s diversity indexes were significant (*P* < 0.05) different between male and female fish. The phylum profile in the intestine of male fish revealed that phylum Proteobacteria was dominant, in contrast to female fish where five phyla Tenericutes, Proteobacteria, Firmicutes, Fusobacteria and Spirochaetes were dominant. The genus profile revealed that genera *Shewanella* and Unclassified bacteria were dominant in male fish, while genus *Mycoplasma* was dominant in female fish.

**Conclusions:**

Our results revealed that the intestinal microbial community of wild largemouth bronze gudgeon was dominated by the phyla Proteobacteria and Tenericutes regardless of the different body weight, but the communities are significant different between male and female fish. These results provide a theoretical basis to understand the biological mechanisms relevant to the protection of the endangered fish species.

## Background

Largemouth bronze gudgeon (*Coreius guichenoti*, Sauvage et Dabry, 1874) is a freshwater fish of the Cyprinidae family, distributed in upstream regions of the Yangtze River. It is of economic importance in China and the largest weight they can reach is 4.0 kg [1, 2]. The fish species is benthic and potamodromous and is typically found in river with torrential flow and they always live in cluster and produce pelagic eggs in flows from March to June every year [3]. However, the species has recently dramatically declined and is close to elimination [4] due to overfishing and construction of hydroelectrically projects, which have blocked the migration routes, causing habitat fragmentation, and losses of spawning grounds and habitat destruction [5, 6].

Over the recent years, the development of omics technologies has boosted our insights on the structure and function of the complex gastrointestinal (GI) microbiota of fish [7–11]. It has been revealed that colonization of microorganisms in the GI tract of fish results in the establishment of a symbiotic relationship between the host and gut microbiota [10, 12], and the fish GI microbiota can contribute to nutrition, health and development [13–15]. In addition, the GI microbiota is important in the defense against adhesion and colonization of pathogenic bacteria [16, 17]. The shaping of the fish intestinal microbiota is a complex process, and a number of factors have been reported to modulate its composition, e.g. host genetics, developmental stage, gut structure, environmental factors, diet and dietary components [10, 12, 18, 19]. However, no information is available on the intestinal microbiota of largemouth bronze gudgeon, a fish species of economic importance in of the Yangtze River.

The ongoing positive growth trend of the ecological protection and species conservation is expected to continue, reflecting the rising demand for largemouth bronze gudgeon rearing in indoor tanks to carry artificial reproduction. Considering the important roles of intestinal microbiota during fish life, the aims of the present study were to elucidate the intestinal bacterial community of wild caught male and female largemouth bronze gudgeon with different body weight from Yangtze River by sequencing of 16S rRNA genes. The evaluation of sex-dependent effects the gut microbiota is of importance to study as less information is available on aquatic animal [20] compared to endothermic animals [21–24]. The results of the present study may be vital for successful propagation of the fish in indoor artificial culture as well as the influence of gender on drug delivery as discussed by Freire et al. [25].

## Results

**Fig. 1:**
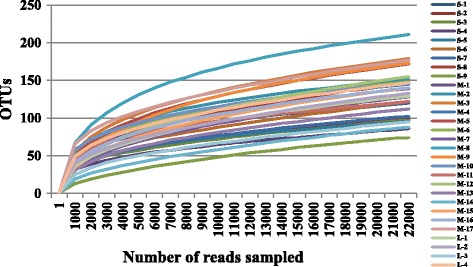
Rarefaction analysis of MiSeq sequencing reads of the 16S rRNA gene in different fish samplings with different body weight. Rarefaction curves at a cutoff level of 3 % were constructed at a 97 % sequence similarity cutoff value. S: small fish, M: medium fish, L: large fish

We obtained 963,883 valid sequences from the 30 fish intestines. After quality filtering and normalization; totally 672,240 high-quality bacterial sequences were obtained, equivalent to an average of 22,408 reads per sample, when representative sequences were classified using RDP classifier. We calculated the number of operational taxonomical units (OTUs), and they were analyzed for each sample with a 97 % sequence similarity cutoff value. Figure 1 shows the rarefaction curve at an OTU definition of 97 % identity. The Good’s coverage of the four samples ranged from 99.70 to 99.86 % (Table 1).

**Table 1:** Number of valid sequence, OTUs, Good’s coverage for 16S rRNA libraries of different fish samplings

Samplings	Valid sequence	OTU (0.03)	Good’s coverage (%)
S-1	34962	123	99.84
S-2	22478	102	99.81
S-3	30409	100	99.83
S-4	26016	88	99.85
S-5	30060	98	99.86
S-6	23191	125	99.74
S-7	33397	105	99.82
S-8	36803	149	99.75
S-9	26295	77	99.83
M-1	44794	142	99.78
M-2	29601	153	99.83
M-3	35898	182	99.73
M-4	31329	148	99.80
M-5	34235	175	99.72
M-6	22424	151	99.77
M-7	43326	131	99.77
M-8	31027	214	99.73
M-9	24136	176	99.72
M-10	22940	149	99.81
M-11	35060	125	99.81
M-12	24712	158	99.70
M-13	26503	115	99.78
M-14	43900	101	99.79
M-15	35472	143	99.74
M-16	25004	143	99.77
M-17	32352	180	99.71
L-1	43437	132	99.78
L-2	45887	136	99.74
L-3	39377	108	99.79
L-4	28858	150	99.75

*OTU* operational taxonomical unit, *S* small fish, *M* medium fish, *L* large fish

### Intestinal microbial community in fish with different body weight

**Fig. 2:**
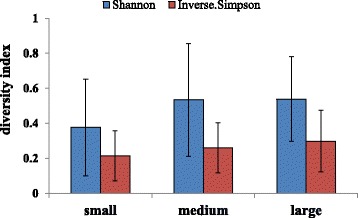
Alpha diversity measures based on average species-level operational taxonomic units (OTUs) of fish with different body weight. Error bars indicate SD

To provide an overview of the sequence reads associated with wild caught largemouth bronze gudgeon intestine, the 30 samples were divided into three groups according to fish body weight: large fish (>2 kg, 4 samples), medium fish (between 1 and 2 kg, 17 samples) and small fish (<1 kg, 9 samples). In small fish, the Shannon and Inverse Simpson diversity indexes were 0.38 ± 0.21 and 0.21 ± 0.14, respectively, while the indexes were somewhat higher, but not significantly (*P* >0.05) different for medium - and large fish (Fig. 2).

**Fig. 3:**
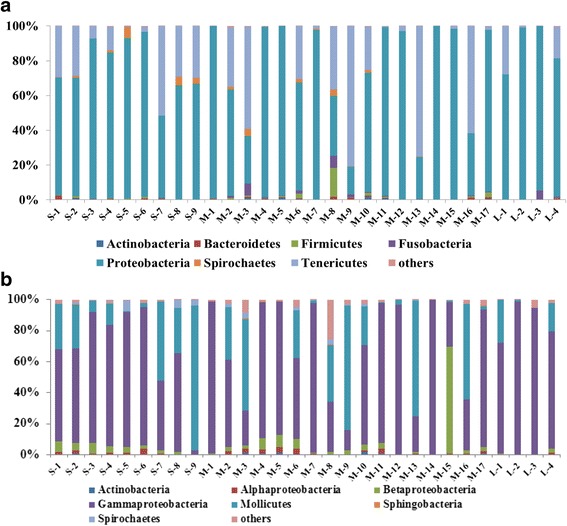
Distribution of average read number among the major phyla (**a**) and major class (**b**) in fish samplings with different body weight. S: small fish, M: medium fish, L: large fish

The bacterial communities of the intestines from all samplings constituted of totally 18 different bacterial phyla, of which seven phyla were dominant, and represented 99.8 % of the entire sequence reads (Fig. 3a). Bacteria within phylum Proteobacteria was dominant in the intestine of wild caught largemouth bronze gudgeon and constituted for 68.4 % ± 29.2 in small fish, and 71.0 % ± 20.8 and 86.3 % ± 10.9 in medium- and large fish, respectively. The abundance of phylum Tenericutes was 28.4 % ± 19.3 in small fish; 24.1 % ± 18.2 and 11.7 % ±10.7 in medium - and large fish, respectively. The remaining five phyla: Actinobacteria, Bacteroidetes, Firmicutes, Fusobacteria and Spirochaetes constituted for less than 2 % of the total bacterial community of all fish. Gammaproteobacteria was dominant; 63.6 % ± 27.4 in small fish; 62.9 % ± 31.9 and 85.0 % ± 13.5 in medium - and large fish, respectively (Fig. 3b).

### Intestinal microbial community in different gender

**Fig. 4:**
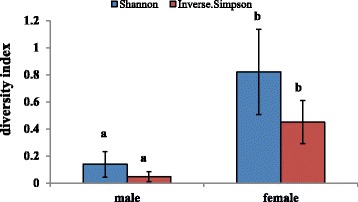
Alpha diversity measures based on average species-level operational taxonomic units (OTUs) of fish with different gender. Error bars indicate SD, a, b indicates significant association (0.005 < *P* < 0.05)

**Fig. 5:**
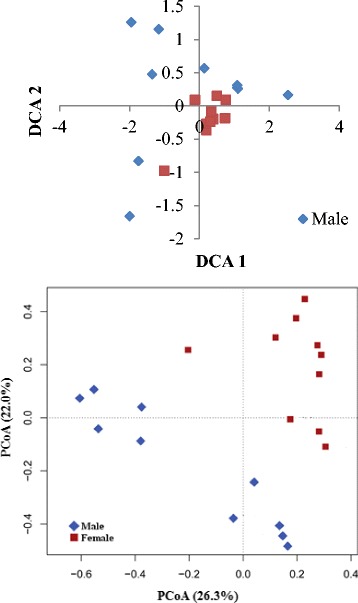
Detrended corresponding analysis (DCA) and principal coordinate analysis (PCoA) based on based on the taxonomic composition (left) and weighted UniFrac distances (right) of fish intestinal samples with different gender

Twenty sexual mature individuals were identified in the present study, 10 male and 10 female, and they were analyzed to compare their microbial community profile. Shannon’s and Inverse Simpson’s diversity indexes in male fish were 0.14 ± 0.09 and 0.05 ± 0.03, respectively, while the indexes were significantly (*P* < 0.001) higher in female fish (Fig. 4). DCA and PCoA ordination respectively based on the microbial compositions and weighted UniFrac distances revealed that male and female fish harbored different intestinal microbial community (Fig. 5).

**Fig. 6:**
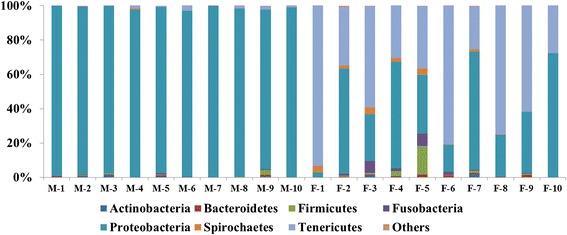
Distribution of average read number among the major phyla in male and female samplings. M: male fish, F: female fish

16S rRNA gene sequence reads analysis revealed that the bacterial community composition was different between female and male fish. In the intestines of male fish, the dominant phylum Proteobacteria constituted 97.6 % ± 1.8 of the microbiota, while the other phyla constituted less than 2.5 % of all reads (Fig. 6). In female fish intestines, phyla Tenericutes and Proteobacteria were dominant, accounting for 52.3 % ± 23.5 and 40.5 % ± 22.8, respectively. Phyla Firmicutes, Fusobacteria, Spirochaetes, Actinobacteria and Bacteroidetes were also identified, and constituted for 2.4 % ± 1.2, 1.9 % ± 1.5 and 1.7 % ± 1.3, 0.4 % ± 0.2 and 0.6 % ± 0.5, respectively (Fig. 6).

### Intestinal genera in fish with different gender

The five most abundant genera in male and female fish intestines are shown in Table 2. The total abundance of these genera relative to the total amount of reads was 91.1 % ± 7.64 for male fish and 87.4 % ± 10.2 for female fish. The dominant genus in male fish was unclassified bacteria and constituted for 34.9 % ± 11.3 of the entire reads with non-significant difference to female fish. In male fish, unclassified bacteria belonging to phylum Proteobacteria and family Enterobacteriaceae were dominant and comprised of approximately 75 % of the bacterial community. Genus *Mycoplasma* (52.3 % ± 24.8) belonging to phylum Tenericutes dominated in female fish intestine, but the relative abundance significantly (*P* < 0.01) decreased to 0.98 % ± 0.35 in male fish. The abundance of genera *Aeromonas* and *Pseudomonas*, both belonging to phylum Proteobacteria were not significant different between male and female fishes. However, the abundance of genus *Shewanella* (phylum Proteobacteria) was significantly (*P* < 0.05) higher in male fish intestine than that in female fish.

**Table 2:** Average relative abundances (% of sequences per treatment) and standard deviation of the most abundant bacteria at genus taxonomy level in fish intestine

Phylum	Genus	Male (mean % ± SD)	Female (mean% ± SD)	Student’s *t*-test *P* value
Tenericutes	*Mycoplasma*	0.98 ± 0.35	52.3 ± 24.8	0.000
Proteobacteria	*Aeromonas*	21.0 ± 12.1	8.2 ± 5.20	0.328
Proteobacteria	*Pseudomonas*	11.1 ± 4.30	9.78 ± 4.07	0.824
Proteobacteria	*Shewanella*	23.1 ± 10.9	0.93 ± 0.46	0.047
	*Unclassified*	34.9 ± 11.3	16.2 ± 3.20	0.143

## Discussion

Previous studies on largemouth bronze gudgeon, a Cyprinidae, have focused on its genetic diversity and stress responds [4], while no information is available on the intestinal microbiota. This topic needs to be evaluated as the intestinal microbiota of endothermic animals as well as fish plays a crucial role to the host [12, 26], and knowledge of the intestinal microbiota and its modulation in largemouth bronze gudgeon may be of importance to understand wild fish viability under cultured conditions as reported for sea bream (*Sparus aurata*) [27].

The non-significant difference in bacterial diversity by the Shannon and Inverse Simpson diversity indexes between fish with different body weight (>2 kg, 1–2 kg and <1 kg), indicate that different fish body weights had no effect on intestinal microbiota of largemouth bronze gudgeon; probably due to similar habitats.

In a study with common carp (*Cyprinus carpio* L.), Li et al. [28] reported a core gut microbiota of Fusobacteria, Proteobacteria, Bacteroidetes and Firmicutes but this is inconsistent with the core gut microbiota: Proteobacteria and Tenericutes of largemouth bronze gudgeon revealed in the present study, although the fish species belong to the same family; omnivores and benthic life. One possible reason for this difference may be that largemouth bronze gudgeon is omnivores but prefer animal food in nature environment contradict to common carp [29]. It is reported that the intestinal microbiota associated with host trophic level, and the intestinal bacterial diversity decrease in herbivores to omnivores to carnivores’ fish species [30–32].

The intestinal bacterial community between male and female largemouth bronze gudgeon was different in the present study, which is in accordance with Iehata et al. [20], revealing difference in both bacterial community and bacterial nutritional enzyme activity between female and male Chilean octopus (*Octopus mimus*). Moreover, endothermic animals studies have also revealed that the gut microbiota composition between gender are different [21–24, 33]. The difference in gut microbiota between sexes, may be hormones associated with each sex that might affect the composition of the gut microbiota [34, 35]. Different feed preference may be another reason for the variations in the intestinal microbiota, as the fish were sampled during different month during the season. In addition, the water temperature may influence the feeding behavior and gut microbiota. However, male and female fish sampled from different sampling times showed no variations within group in the present study. In their review, Freire et al. [25] discussed the influence of gender on gastrointestinal physiology and drug delivery. Whether the results of the present study may be of importance for drug delivery in fish is not known and merits investigation.

Intestinal bacteria can serve the host in two ways: they can represent a nutrient source and/or contribute with enzymes that may improve host digestion [13, 36]. Previous studies, revealed that some strains of genera *Aeromonas* and *Pseudomonas* produced amylase efficiently in freshwater fish, and this finding suggest that the intestinal microbiota may play an important role to the host [37]. In the present study, the genera *Aeromonas* and *Pseudomonas* were dominant in male and female largemouth bronze gudgeon, but no significant difference was revealed. Whether these bacteria species contribute to the fish nutrition is a topic for further investigation.

It is of interest to note that the most dominant genus in male and female largemouth bronze gudgeon was unclassified bacteria (phylum Proteobacteria and family Enterobacteriaceae) and *Mycoplasma* (phylum Tenericutes), respectively, which is different to the findings reported for other freshwater fish species [28, 38]. Ringø et al. [9] showed that the abundance of Enterobacteriaceae were affected by protein sources. This may be of interest as some members of Enterobacteriaceae have been reported to benefit metabolic activity; saccharolytic and utilizing acetate, while other members of the family are potentially opportunistic pathogens [39]. Whether the protein sources for male and female fish development were different and what’s the function of these unclassified bacteria merit further investigation. Genus *Mycoplasma* is reported as pathogens for human, animals and plants [40]. However, Holben et al. [41] detected a novel *Mycoplasma* phylotype which comprised for approximately 96 % of the total microbes in the distal intestine of wild Atlantic salmon (*Salmo salar* L.), which were substantially different from those indicated in pen-raised salmon from Scotland and Norway. Moreover, the authors speculate the *Mycoplasma* species could utilize cytoplasmic secretions from the host and produce lactic and acetic acids which subsequently utilized by other bacteria in wild salmon intestine. Considering the significantly (*P* <0.01) increase in abundance of genus *Mycoplasma* in female largemouth bronze gudgeon, some special metabolic activity may exist in female fish.

Genus *Shewanella*, which have been isolated from marine environments [42], were detected at a significantly (*P* <0.05) higher abundance in male largemouth bronze gudgeon in the present study. This finding may be of importance as several strains of genus *Shewanella* have previously been reported to produce polyunsaturated fatty acids [43]. As the predominant genera in largemouth bronze gudgeon were more similar to Atlantic salmon than common carp, similar family as largemouth bronze gudgeon, this topic requires further evaluation.

## Conclusion

The present study revealed that the intestinal microbial community in wild largemouth bronze gudgeon sampled from the Yalong River, had a core intestinal microbiota: phyla Proteobacteria and Tenericutes regardless of different body weight. In addition, it is of interest to notice that difference occurs in the intestinal bacterial community between male and female fish, which may due to the various metabolic activities in male and female fish. The results of the present study increase our understanding of the microbial ecosystem diversity in an endangered fish species. Furtherly, understanding the associations between the structure and function of intestinal bacterial communities and body ecosystem parameters is important for determining the fish’s physical condition, and such information will also contribute to optimize breeding regimes and improve the health of endangered fish species in captivity.

## Methods

### Fish of the study

During April, June and fall (October-November) 2013, 30 wild largemouth bronze gudgeon were sampled from the Yalong River, the upper reaches of the Yangtze River (Yanbian county). The fish were placed in oxygen filling box, transported to the laboratory on ice, and measured for body length and body weight. Male and female fish were identified according to gonadal development.

### Sample collection

All fish were anesthetized with an overdose of MS 222 (3-Aminobenzoic acid ethyl ester methanesulfonate, Sigma, Germany). The exterior surfaces were swabbed with 75 % ethanol before dissection of the whole intestine using sterile instruments (scissors and tweezers). The intestine of each individual fish was dissected out, and similar weight (about 0.2 g) of foregut, midgut and hindgut from each individual was collected and pooled together into a sterile tube as a single sample as described previously [38]. The individual intestinal contents were homogenized by vortexing briefly.

### DNA extraction

DNA preparation was performed by incubating intestinal homogenates in 1 ml lysis solution (30 mmol l^−1^ EDTA, 10 mmol l^−1^ Tris-HCl, 0.5 % sodium dodecyl sulphate (SDS), 0.1 mg proteinase K, 0.05 mg RNase A) overnight at 55 °C, followed by standard phenol/chloroform extraction as previously described [44]. DNA solution was stored at −20 °C until further use.

### MiSeq sequencing of bacterial 16S rRNA gene amplicons

Liu et al. [45] reported that the V4 region shows few biases for different bacterial taxa, and the region is considered to yield accurate taxonomic information. Therefore, the V4 region of 16S rRNA gene was used in the present study to assess the fish intestinal bacterial community. PCR amplifications were performed in triplicate with the bacterial primer sets 515 F/806R [46], PCR products were purified using Agencourt® Ampure® XP beads (Beckman, CA, USA) according to the manufacturer’s instructions. The purified DNA was then used as a template to perform a second PCR using the same primer sequences and the protocol, but the reverse primer sequence included appropriate adapters and different barcodes for the identification of samples. PCR products were visualized on 1 % agarose gels and negative controls were performed each time to ensure that no contamination had occurred.

PCR products were quantified using the PicoGreen dsDNA Assay kit (Invitogen, Carlsbad, CA, USA), equally combined and followed by gel purification using a QIAquick Gel Extraction Kit (Qiagen, CA, USA), and then re-quantified by PicoGreen. The prepared DNA library was then sequenced using the MiSeq platform (Illumina, CA, USA) following the manufacturer’s instructions. Quality filtering and processing of sequence reads were conducted on Galaxy pipeline (http://zhoulab5.rccc.ou.edu:8080/root) as described previously [47]. An OTU table was generated using the Uparse clustering method (97 % cutoff), and all samples were rarefied to the same sequencing depth by resampling OTUs prior to downstream analysis.

### Statistical analysis

The representative sequence of each OTU was used for taxonomy assignment using Ribosomal Database Project (RDP) classifier [48]. In order to compare the bacterial communities, good’s coverage and alpha-diversity indices were calculated according to the procedures described by Caporaso et al. [49]. Alpha- measurements were applied to describe species composition in one specific habitat and the differentiation among habitats, respectively according to Peter et al. [50]. Detrended correspondence analysis (DCA) and UniFrac distance-based PCoA analyses were also performed to visually depict the differences between male and female fish. One way ANOVA and two-tailed Student’s *t*-test was used to assess the differences of intestinal bacterial communities between male and female fishes. Statistical analyses were performed with the software PASW SPSS 18.0 (IBM, USA) and R 3.2.0 (Lucent Technologies, USA) package.
